# Hippocrates of Kos (460-377 BC): The Founder and Pioneer of Clinical Medicine

**DOI:** 10.7759/cureus.70602

**Published:** 2024-10-01

**Authors:** Nikolaos A Kostakopoulos, Themistoklis C Bellos, Stamatios Katsimperis, Lazaros Tzelves

**Affiliations:** 1 First Department of Urology, Metropolitan General Hospital, Athens, GRC; 2 Second Department of Urology, Sismanogleio General Hospital, National and Kapodistrian University of Athens, Athens, GRC

**Keywords:** hippocrates, hippocratic oath, historical vignette, holistic medicine, medical ethics

## Abstract

Hippocrates was the first physician in history to establish medicine as a science and to suggest the boundaries of physicians' behavior towards their patients. The Hippocratic Oath is applied in many healthcare systems worldwide as an ethical guide for doctors graduating from medical school. It determines modern medicine’s most significant values, such as physicians' specialization to avoid harm and respect patients’ privacy. In this study, we present Hippocrates' contributions to clinical medicine and his innovative ideas for the prevention, diagnosis, and treatment of disease. We also analyze his achievements in the development of the main concepts of several medical specialties, such as neurology with his approach to the treatment of epilepsy, surgery with his techniques of antisepsis, urology with his theory on stone disease, orthopedics, and acute medicine, as well as their application in modern healthcare. We have conducted a review of the available literature from PubMed and Google Scholar databases.

## Introduction and background

One of the most well-known physicians of ancient Greece and possibly of all time, Hippocrates was born on the Aegean island of Kos during the fifth century, which was also famous as the golden age of Pericles. During the fifth century, a plethora of great personalities lived in Athens and contributed significantly in many fields of modern science and art [1,2].

Being inspired by the innovative spirit of his age he was able to study medicine without religious prejudice but rather as a science that prevents, predicts, diagnoses, and treats diseases that are caused by environmental or hereditary factors [2].

He was also successful in developing the basic aspects of many medical specialties, such as neurology, acute and respiratory medicine, and surgery and urology [1,3-7]. Hippocrates was greatly influenced by the theory of the fifth-century philosopher Empedocles that nature was made of the four elements that are water, earth, fire, and wind, and thus the human body also consisted of four fluids or "humors" - black bile, yellow bile, phlegm, and blood - as well as four elemental conditions (hot, cold, dry, and moist). Health was associated, according to the great physician, with a balance of all these elements [5]. Moreover, Hippocrates' medicine was influenced by many epidemics that roamed ancient Greece during his lifetime, such as the typhoid fever of Athens and Thessaly during the Peloponnesian War. These epidemics helped him develop his techniques on antisepsis and prevention of diseases [5].

Furthermore, Hippocrates managed to combine his principles on physical examination and his ideals on the ethical rules of practicing medicine by establishing the Hippocratic Oath [2,8-10]. This is a one-page oath given by doctors when they finish medical school and is used by many healthcare systems as guidance on professional behavior towards their patients. It has been revised several times, with the most important update introduced in 1948 by the World Medical Association [9].

## Review

Main contributions to medicine

By studying the collective work of Hippocrates we can find references for his contributions in clinical medicine. Such crucial phrases are the aphorisms, which consist of seven parts with short medical truths. They emphasize the importance of experienced physicians performing specialized procedures and surgery, and make suggestions on lifestyle changes to improve health [1].

*Prevention of Disease*

Another important aspect of Hippocrates' works that is widely applied in 21st-century medicine is the prevention of disease. The phrase “Κάλλιον το προλαμβάνειν του θεραπεύειν,” which means that it is better to prevent than to treat a disease, was the cornerstone of his teachings and is based on the observation that healthy Mediterranean diet and daily moderate physical activity can prevent disease. The ancient Greeks believed that all maladies started from the gut and that walking was the best available medicine [2].

*The Hippocratic Oath*

The Hippocratic Oath summarizes the main aspects of Hippocrates' way of thinking and most of its advice can be applied in modern-day medicine. The phrase “I will not harm” emphasizes the obligation of the physician to treat patients according to their specialized abilities, in order to help them and not cause them harm by getting involved with unethical remedies or unknown crafts, for example, surgical procedures for nonspecialized doctors [1,8,9,11].

Moreover, Hippocrates underlines the necessity of patients’ privacy protection by physicians, who are urged to be discreet with their patients' problems and not make them public. It's crucial for this ethical rule to be applied in modern healthcare systems, where there is a rising necessity to safeguard patients' data on online databases and social media platforms [8,9,11].

*Holistic Approach*

One of the most significant innovations of the founding father of clinical medicine was the holistic approach for the diagnosis and treatment of disease. This approach is based on the assumption that the human body is a sum of many parts that function in harmony and that if one part is ill, the balance will be affected and the whole person will suffer. Hippocrates considered that patients consisted of body, mind, and spirit and this is also the modern physicians' approach when treating a disease [2].

Contributions of Hippocrates to medical specialties

*Urology*

The work of Hippocrates contributed greatly to two sections of the urologic specialty. These were uroscopy, which is the macroscopic observation of urine, and the causes of lithiasis of the kidneys [1].

Contributions to stone disease: By observing the patients’ urine it was possible for the Greek physician to describe the sedimentation of sand and gravel which accumulate to form “lithous” the Greek word for stones [1]. Hippocrates also attributes stone formation to the quality of drinking water and to inflammation of the bladder neck, which causes urine stasis. This fact has been evidently proven in current urological practice since benign prostatic enlargement and chronic prostatitis can cause obstruction of urine outflow, urine stasis, and eventually bladder stone formation.

Stone formation is mentioned to be more frequent in male children due to longer, narrower, and more angular urethra than in females. Added to this, Hippocrates was one of the first physicians to suggest an increase in fluid, especially water, intake to prevent lithiasis (stone formation), which is still the main preventive measure to avoid stone formation today [1].

Contributions to uroscopy and urine analysis: Apart from this, Hippocrates studied several features of urine (uroscopy) in association with acute and chronic disease. Presentation of blood in the urine (hematuria, from the Greek word “haema” which means blood) may indicate an injury of the renal vessels or even ischemic necrosis, while colorless urine was considered a bad sign associated with a systemic disease, which may be attributed to the failure of urine concentration in chronic kidney disease [1]. 

A crucial phrase from the Hippocratic Oath “I will not use the knife, not even, verily, on sufferers from stone, but I will give place to such as are craftsmen therein” recognizes the importance of specialization in medicine and can be acknowledged as the first reference to urology as a surgical specialty or “craft” [1,9].

Acute and Respiratory Medicine

The most important value that a physician should follow, according to Hippocrates, is a meticulous clinical examination, which includes careful observation of the patient and detailed history taking of his symptoms and signs [3].

In emergency situations, Hippocrates and his students gave great attention to the patient's breathing pattern, pain, and look of their face which is called the Hippocratic face and is associated with serious conditions that could lead to death [3].

In many of his works, acute respiratory conditions such as pneumonia, pleurisy, thoracic empyema, and airway obstruction, as well as their treatments are described [7]. The physical examination of patients with respiratory symptoms should include a thorough observation of the whole body with a holistic approach because a disease of the lungs could also systematically affect other parts of the body [2,7]. Several of Hippocrates' suggestions for the clinical examination and treatment of respiratory conditions can still be applied today, including increasing hydration, mobilization, and using tubes to inhale vapors, like modern-day inhalers. Invasive treatment of other acute respiratory conditions was also described, such as cauterization and paracentesis of thoracic empyema [7].

Neurology

Hippocrates, as previously mentioned, considered the human body to be a sum of many parts that co-exist in a harmonic balance. One of the most fundamental parts of the body according to his writings was the brain. He claimed that the brain was the organ responsible for intelligence and consciousness [4]. Conditions like hemiplegia, paraplegia, apoplexy, and epilepsy are often analyzed in his work and a large percentage of modern-day neuroscience references his advancements in the specialty of neurology [4].

The ancient Greek physicians’ treatise on the sacred disease, which was the name given to epilepsy before his time, contributed greatly to diminishing any theories of the divine origin of the disease and categorizing it as a relatively common brain disorder [12].

Hippocrates considered the accumulation of black bile and mucus in the veins of the brain to be the cause of epilepsy, which he thought was a hereditary disorder. He proposed a conservative treatment with herbal potions and in serious cases surgical intervention with trepanning, which means open brain drilling, a precursor of modern-day neurosurgery. Trepanation was developed in modern-day neurosurgery under the term craniotomy for the treatment of epidural and subdural hematomas, as well as for gaining surgical access for most neurosurgical procedures [12].

Surgery and Orthopedics

Hippocrates was not only the founding father of clinical medicine but also laid the foundation of surgery. He clarified that it was a different specialty and only physicians specifically trained in surgical techniques should practice them [1,8,9]. His works described surgical techniques of general surgery, urology, orthopedics, and neurosurgery [1,12,13].

The famous physician had studied many organs of the human body and described their association with the four humors (blood, yellow bile, black bile, and phlegm) [5]. For example, the clearance of the black bile was attributed to the spleen, a visceral spongy organ [6]. Furthermore, surgical interventions such as phlebotomy and local cauterization were suggested in cases of splenic hardening and splenic abscess [6].

Contributions to antiseptic techniques: Apart from this, the works of Hippocrates include many references to antisepsis, anesthesia, and abdominal surgery. Some surgical interventions described are the puncture of a liver abscess, without injuring the liver capsule, and the drainage of ascitic fluid collection by a periumbilical incision. It is even suggested that a metallic drain is left in place, but also the importance of avoiding injury to visceral organs and the hypogastrium [13].

It is highlighted that the surgeon should have an organized medical bag that includes a large variety of surgical instruments, swabs, catheters, magnifying glasses, and instruments to cauterize wounds. The use of the plant mandrake (Greek: Mandragoras) is mentioned as a general anesthetic because it contains scopolamine, combined with other sedative herbs [13].

Most importantly, an antiseptic protocol was followed by Hippocrates and his followers before every procedure. This included cleaning the surgical field with boiled water, salt, seawater, and natural perfumes [13]. Ligation of vessels was performed with cauterization and sutures made of animal intestines, which are still being used today in their improved forms. The skin of the incision was sutured or stapled and the surgical wound was cleaned with wine and other antiseptic ointments to prevent infection. Lastly, the wound was covered with clean pads and bandages. All the above measures have withstood the evolution of science and technology and are still being used in modern surgical principles [13].

Contributions to orthopedics: Last but not least, Hippocrates dedicated a whole volume of his works to orthopedics and traumatology, where he introduced innovative techniques to reduce bone fractures and joint dislocations and suggested treatments for osseous infections and gangrene. His techniques and ideas have been evaluated and applied in modern treatments of skeletal and spinal diseases [14].

## Conclusions

Hippocrates of Kos is considered the founding father of clinical medicine and surgery. His innovative studies have introduced most modern-day specialties, such as surgery, urology, neurology, and acute medicine, and have set the ethical rules for practicing medicine by physicians with the Hippocratic Oath. His contributions include the basic principles of antisepsis and lifestyle changes for the prevention of disease, as well as the holistic approach when examining a patient. The knowledge of Hippocrates' ideas and their application with necessary advancements have greatly improved the diagnosis and treatment of diseases.
